# A Prospective Randomized Study to Compare Standard to Reverse Insertion Techniques of Classic Laryngeal Mask Airway (LMA) in Pediatric Patients

**DOI:** 10.7759/cureus.95079

**Published:** 2025-10-21

**Authors:** Atit Kumar, Prashant Mishra, Shuchi Nigam, Usha Shukla, Kuldeep Sahay

**Affiliations:** 1 Anesthesiology, Uttar Pradesh University of Medical Sciences (UPUMS), Etawah, IND; 2 Anesthesiology, Rama Medical College and Hospital, Hapur, IND; 3 Anesthesiology and Critical Care, Uttar Pradesh University of Medical Sciences (UPUMS), Etawah, IND

**Keywords:** complications, general anaesthesia, insertion, laryngeal mask airways, paediatrics

## Abstract

Introduction

The laryngeal mask airway (LMA) has emerged as a viable option for managing airways. It is an alternative rescue technique for both anticipated and unanticipated difficult airways in both elective and emergency settings.

Methods

In our study, 120 patients were divided into two groups: Group S (n=60), standard technique for classic LMA (c-LMA) insertion, and Group R (n=60), reverse technique for classic LMA insertion. The number of insertion attempts was recorded. Insertion was considered easy if there was successful placement on the first attempt with or without maneuvers. We also noted the total time for the successful placement of the airway.

Results

In Group S, successful LMA placement on the first attempt was done in 93.3% of the patients, compared to 86.7% of the patients in Group R, though it was not statistically significant (p=0.5265). The mean time required to secure the airway in Group S was 16.28±1.93 seconds, whereas in Group R, it was 18.40±2.92 seconds (p<0.0001). The difference in hemodynamic parameters was nonsignificant (p≥0.05). In Group S, incidences of complications were lower compared to Group R, but it was not statistically significant (p>0.05). Irrespective of the technique used, the failure rate was zero.

Conclusion

Considering the shorter insertion time, along with the lower complication rates, the standard technique may be regarded as the preferred method of LMA insertion over the reverse technique.

## Introduction

A wide range of abilities and specialized knowledge is needed for effective airway control. It requires certain skills to use the optimal airway management strategy and operate a variety of airway devices in order to predict challenging airways and create an airway management plan. Failure of airway control can result in consequences such as dental trauma, pulmonary aspiration, airway trauma, anoxic brain injury, cardiac arrest, and death [1].

The field of supraglottic airway devices (SGADs) has had a remarkable evolution since the invention of the traditional laryngeal mask airway (LMA), and SGADs are currently often utilized in clinical anesthesia. In order to assist the efflux of gastric contents and to facilitate the insertion of a gastric tube, more recent SGADs come equipped with an integrated drainage channel [2].

Although LMA placement is even more challenging in children, it is also frequently utilized in the operating room for airway management under anesthesia [3]. The size of the LMA used in newborns and children is a smaller, scaled-down version of the adult LMA because of the physical differences between the two groups [4,5]. Although several methods for LMA insertion have been proposed, only a few of the successful and less complicated LMA insertion techniques have been used in the past [6,7]. Researchers have looked into a variety of LMA insertion approaches, including the reverse and the conventional methods [8].

In Brain's insertion technique (conventional technique), the deflated airway was inserted with its lumen pointing forward until resistance was felt. The cuff is typically empty when using the conventional method of LMA insertion. Despite its popularity, this method has some disadvantages. The primary issue occurs when the LMA tip folds up against the posterior wall of the pharynx [9]. Passing the LMA cuff through the posterior arch of the throat is facilitated by slightly inflating it, which increases the likelihood of successful insertion. Using the index finger to drive the LMA into place involves a lot of force, which could cause the patient's teeth to injure the guiding finger. This would frequently result in several insertion attempts, airway damage, and an improper seal.

There are times when insertion is difficult, and numerous authors have proposed insertion methods that are different from Brain's typical approach [10]. The "reverse technique," which involves inserting the LMA with the cuff facing the palate and later turning it 180 degrees, is one such technique. The rotational technique has been shown to yield significantly superior outcomes as it helps bypass the bulky tongue and epiglottis with minimal resistance in pediatric airway management. Without bending the tip, the LMA glides along the posterior pharynx and avoids the structures in the anterior pharynx.

Recently conducted studies on different methods of insertion have been done mostly in the adult population. Because of the limited evidence, we conducted this study with the aim of comparing the ease of classic LMA (c-LMA) insertion using the standard and reverse techniques in two groups with respect to the number of attempts required for successful insertion and the time taken to secure a successful airway. We also compared the hemodynamic parameters, the incidence of complications, and the failure rate of classic LMA insertion in both techniques.

## Materials and methods

This prospective randomized study was carried out from December 2023 to November 2024 after approval from the Institutional Ethics Committee of the Pradesh University of Medical Sciences (ethical clearance number: 67/2023-24) and registered with Clinical Trials Registry India (CTRI) (registration number: CTRI/2023/12/060734). On the basis of a previous study conducted by Shyam and Selvaraj, the sample size was calculated considering an alpha error of 0.05, a confidence interval of 95%, and a power of 80%; the sample size came out to be 54 cases in each group [11]. Taking into account a 10% dropout rate, we have taken a total sample size of 120 (60 patients in each group). The patients aged 5-10 years, of either sex, weighing 15-30 kg, with American Society of Anesthesiologists (ASA) I and II, and scheduled for elective surgeries under general anesthesia, with a duration of less than one hour, were included in the study. It is free to use and does not need any license [12]. The patients not ready to participate in the study and with a difficult airway were excluded from the study. Randomization was done using a computer-generated random number table, and group allocation was done using the sequentially numbered sealed opaque envelope technique. The anesthesiologist performing LMA insertion and the observer recording outcomes were not blinded to the group allocation.

All the patients who met the inclusion and exclusion criteria underwent a thorough pre-anesthetic checkup. Parents/guardians of the participants were informed thoroughly about both the techniques, and written informed consent was obtained from them. The patients were divided into two groups: Group S received the standard technique of LMA insertion, while Group R received the reverse technique.

The patients were kept nil per os according to the nothing by mouth (NPO) guidelines before surgery. In the operation theatre, ASA standard monitoring devices including electrocardiography, noninvasive blood pressure (NIBP), peripheral oxygen saturation (SpO_2_), temperature probe, and bispectral index (BIS) monitors were attached to the patients, and the baseline hemodynamic parameters, heart rate (HR), systolic blood pressure (SBP), diastolic blood pressure (DBP), mean arterial pressure (MAP), and SpO_2_, were recorded. The patients were premedicated with injection glycopyrrolate 0.01 mg/kg, injection midazolam 0.05 mg/kg, and injection fentanyl 2 µg/kg intravenously (IV), and preoxygenation was done for three minutes. The patients were then induced with injection propofol 2 mg/kg IV, followed by muscle relaxation with injection vecuronium 0.1 mg/kg IV. After proper muscle relaxation and appropriate depth of anesthesia were achieved, the LMA was inserted by an experienced anesthesiologist. The technique of LMA insertion was according to the group allocated.

**Figure 1:**
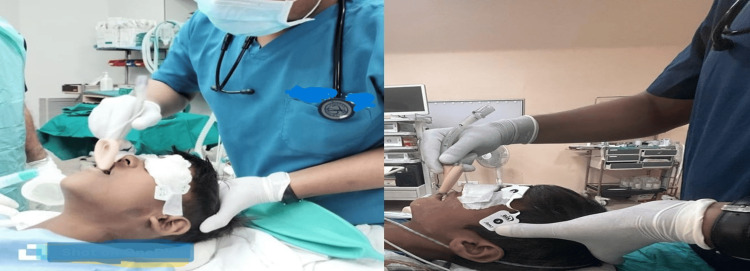
Standard and reverse techniques of classic LMA insertion LMA: laryngeal mask airway

In Group S patients, a classic LMA was introduced with its concavity facing toward the mandible. Then, it was pushed posteriorly while advancing along the hard palate, soft palate, and posterior pharynx and placed in its final position. In group R patients, a classic LMA was inserted with its concavity facing toward the hard palate. Upon reaching the pharynx, the device was rotated into its final position to enable positive pressure ventilation (Figure 1).

The appropriate placement of the classic LMA was confirmed by the presence of a square wave capnography, bilateral chest auscultation, visible chest wall movement, and the absence of an audible leak at a peak airway pressure (PAP) of ≥20 cm H_2_O during manual ventilation. The total time taken for successful placement was recorded, which did not include the confirmation steps. A maximum of three attempts was permitted for each technique. Failure to achieve successful placement within three attempts was considered a failure, and airway management was subsequently performed using an alternative device. The number of insertion attempts was documented. Insertion was considered easy if there was successful placement in the first attempt with or without maneuvers. If effective placement was achieved after more than one attempt, the insertion was deemed challenging (considered a difficult insertion). With the APL valve closed at >20 cmH_2_O and the O_2_ flow maintained at 3 L per minute, the attending anesthesiologist assessed the oropharyngeal leak pressure (OLP) after verifying that the traditional LMA was positioned correctly.

The ventilation was improved by a number of manipulations, including chin lift, jaw thrust, head extension, neck flexion, and moderate advancement, if the leak happens at a pressure of less than 20 cmH_2_O. If the air leak continued after the manipulations, the classic LMA was reinserted using the same method. We noted the total time for the successful placement of the airway. Hemodynamic variables were noted at zero minutes (baseline), one minute, three minutes, and every five minutes after the successful placement of the airway for 15 minutes, after which the hemodynamic variables were measured every 15 minutes until 60 minutes. Anesthesia was maintained with 33% oxygen, 66% nitrous oxide, and 0.2%-0.8% isoflurane, maintaining BIS values between 40 and 60. Adequate muscle relaxation was maintained with intermittent doses of injection vecuronium (0.02 mg/kg IV). At the end of the surgical procedure, anesthesia was discontinued, and muscle relaxation was reversed with injection neostigmine 50 µg/kg IV plus injection glycopyrrolate 10 µg/kg IV. The device was removed after the deflation of the cuff with gentle oral suctioning, with the patient still in a deeper plane of anesthesia, and oxygen maintained by face mask. Complications such as blood staining of the LMA, sore throat, hypoxia (SpO_2_ of ≤90%), and laryngospasm were recorded.

## Results

**Figure 2:**
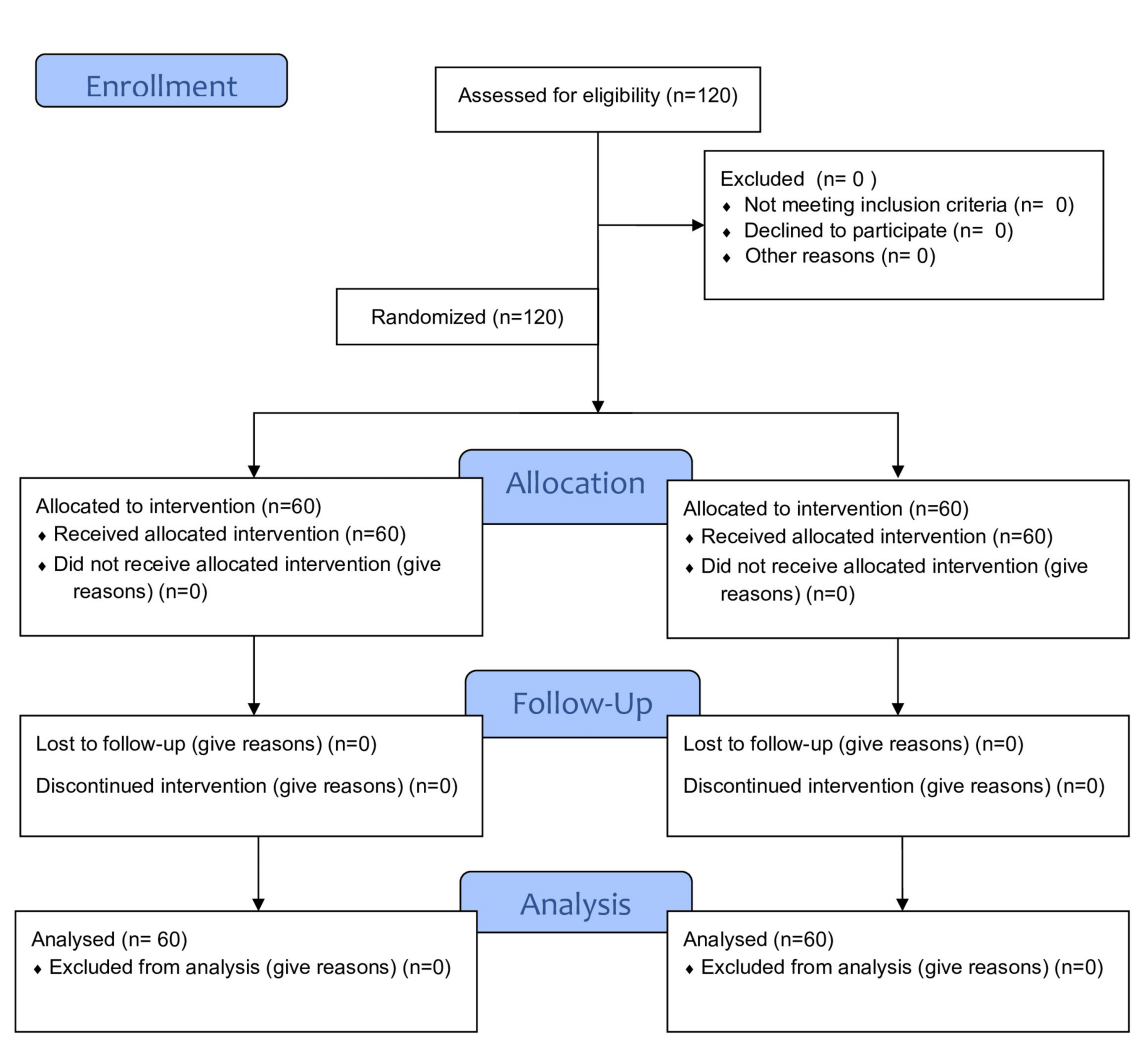
Consort flow diagram

All 120 patients assessed for eligibility were enrolled in the study and underwent randomization. Total patients allocated to receive anesthesia were 60 for each group; none of the patients were lost to follow-up or discontinued from the study (Figure 2). The demographic variables were comparable (p>0.05) between the two groups (Table 1).

**Table 1:** Comparison of the demographic profile of the patients between the studied groups Data expressed as mean and standard deviation (SD) is analyzed by an unpaired Student's t-test. Data expressed in numbers is analyzed by the chi-square test. P-value<0.05: statistically significant n, number of patients in the group; ASA PS, American Society of Anesthesiologists Physical Status

Characteristics		Standard (n=60) (mean±SD)	Reverse (n=60) (mean±SD)	P-value
Age (years)		7.87±1.58	8.13±1.51	t=0.9215; p=0.3587
ASA PS	I	70%	73.3%	X=0.1642; p=0.6854
	II	30%	26.7%	
Weight (kg)		22.27±3.24	22.43±3.49	t=0.2603; p=0.7952
Height (cm)		111.07±6.18	110.50±7.63	t=0.4497; p=0.4497

**Figure 3:**
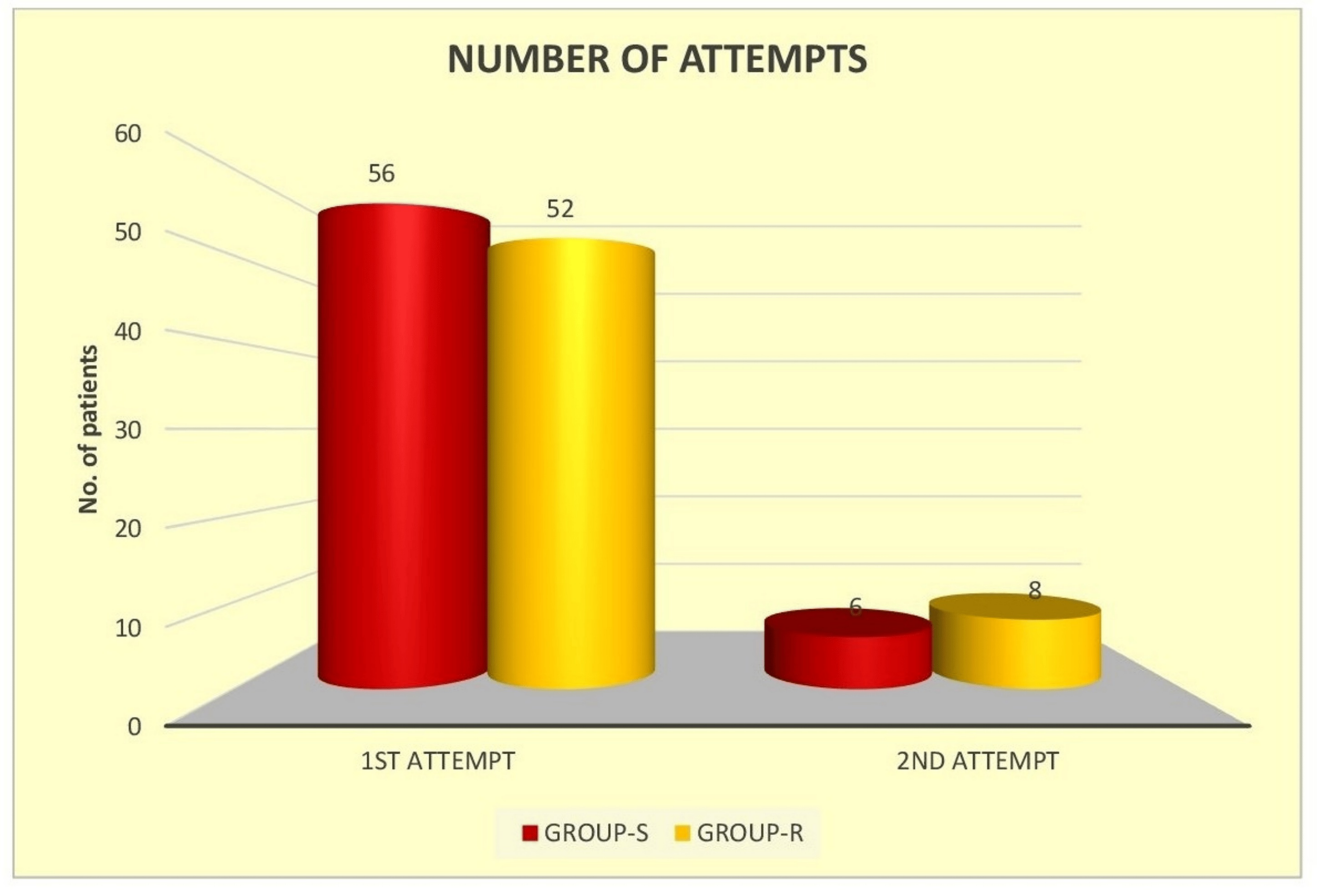
Comparison of the number of attempts between the study groups

In Group S, 56 out of 60 patients (93.3%) achieved successful LMA placement on first attempt, compared to 52 patients (86.7%) in Group R. The need for a second attempt was observed in four patients (6.7%) in Group S and eight patients (13.3%) in Group R (Figure 3). Although Group S showed a slightly higher first-attempt success rate, the difference was not statistically significant (χ² with Yates correction is 0.8333; p=0.36131). The overall success rate was 100% in both groups.

The mean time required to secure the airway in Group S was 16.28±1.93 seconds, whereas in Group R (reverse insertion), it was 18.40±2.92 seconds, which was statistically significant (t=4.692; p<0.0001), indicating that the standard insertion technique allowed for a significantly faster airway placement compared to the reverse technique in pediatric patients (Table 2).

**Table 2:** Comparison of total time taken to secure airway between the groups Data is analyzed by an independent t-test; p-value<0.05: statistically significant *The result (p-value) is highly significant

Variable	Group S (n=60)	Group R (n=60)	P-value
Total time to secure airway (seconds)	16.28±1.93	18.40±2.92	t=4.692, p<0.0001*

Baseline heart rate and intraoperative heart rates were comparable among the groups. The comparison of mean arterial pressure (MAP) between Group S and Group R at various time intervals showed that there was no statistically significant difference at any measured time point. Overall, the MAP was well-maintained and comparable between the two groups throughout the observation period (Table 3).

**Table 3:** Comparison of mean arterial pressure (MAP) between Group S and Group R at different time points Data is analyzed by an independent t-test; p-value<0.05: statistically significant

Mean MAP at time (minutes)	Group S	Group R	P-value
0 minutes	73.2±2.4	73.53±1.5	t=0.9032; p=0.3683
1 minute	72.85±2.4	72.91±2.04	t=0.1648; p=0.8694
3 minutes	73.72±2.99	73.49±1.95	t=0.4904; p=0.6248
5 minutes	73.98±3.53	73.55±1.81	t=0.8396; p=0.4028
10 minutes	73.97±3.53	73.54±2.04	t=0.8170; p=0.4156
15 minutes	74.6±3.08	73.69±1.81	t=1.97; p=0.0508
30 minutes	74.3±3.55	73.57±1.94	t=1.3977; p=0.1648
45 minutes	73.94±3.02	73.34±1.94	t=1.2948; p=0.1979
60 minutes	74.13±3.13	73.43±1.96	t=1.4682; p=0.1447

The blood staining of the device occurred in four patients (6.7%) in Group S and six patients (10.0%) in Group R. Sore throat was reported by six patients (10.0%) in Group S and eight patients (13.3%) in Group R.

## Discussion

Our study evaluated and compared the ease of classic LMA insertion through the standard or reverse techniques, considering the number of attempts and total time taken to secure a successful airway. In this study, the success rate of LMA insertion in Group S with first and second attempts was 93.33% and 6.67%, respectively, whereas in Group R, these values were 86.67% and 13.33%, respectively. There was no need for a third attempt in both groups. Successful placement in the first attempt was slightly higher in Group S (93.33%) compared to Group R (86.66%); this difference was statistically insignificant (p=0.36131). Despite lacking statistical significance, the observation carries clinical importance in the pediatric population, where every second is crucial, and failure on the first attempt can quickly escalate into a difficult airway situation.

Consistent with our study, Karami et al. conducted a randomized controlled trial to study the comparison of the standard technique to the 180-degree rotation technique for classic LMA insertion in 68 patients [13]. The success rate of classic LMA insertion in the first attempt was 92.2% in the standard group compared to 67.6% for the reverse group. The second-attempt success rates for the standard and reverse methods were 8.8% and 32.4%, respectively. The difference in success rate was statistically not significant (p=0.016). A similar study was conducted by Salman in which the success rate of c-LMA insertion in the first attempt was 98% in the standard group compared to 92.5% for the reverse group [14]. The second-attempt success rates for the standard and reverse methods were 4% and 6.5%. respectively, and a third attempt was needed in 1% of the patients in the 180-degree group. However, the success rate was statistically insignificant (p=0.042). Comparable results were seen in the studies conducted by Kumar et al. [15], Prasad et al. [16], and Haghighi et al. [17], in which the first-attempt success rates were higher in the standard group in comparison to the reverse group. Contradictory results were found in a study by Keerthana et al. in which the insertion success rate in first attempt was 85% and in second attempt was 11.7% in the standard group, compared to the reverse group in which the first attempt success rate was 91.7% and the second attempt success rate was 5%, and the third attempt was needed only in standard technique in 1.7% [18]. Success rate was statistically not significant (p=0.56). Similarly, Farbood et al. showed a substantial difference in success rate and problems of the standard approach and the 180-degree rotation method for laryngeal mask insertion in patient airway management [19]. The classic group's first, second, and third attempts at LMA insertion had success rates of 86.3%, 93.5%, and 94.2%, respectively. In contrast, the 180-degree group's first and second attempts had success rates of 98.6% and 100%, respectively, and no third attempt was required. There was statistical significance in the success rate (p=006). This difference may be attributed to differences in patient profiles, the specific LMA devices employed, or the familiarity and skill of the anesthetist with a given insertion method.

In our study, the total time taken to secure the airway in the standard group was shorter (16.28±1.93 seconds) than that in the reverse group (18.40±2.92 seconds), and the mean difference was statistically significant (p=0.0001). The longer mean duration of LMA insertion in the reverse group could be attributed to the folding of the LMA tip during its rotation. Balaji and Jayaprakash conducted a study for the comparison of the insertion technique of classic laryngeal mask airway regarding the ease of insertion and complications, and they found similar results [20]. The mean LMA insertion time of the standard technique and the reverse technique was 34.28±10.20 seconds and 69±48.49 seconds, respectively. The mean difference was statistically significant (p=0.0001). Salman conducted a comparative study of the standard technique with the 180-degree rotation technique for classic LMA insertion in adult patients [14]. In addition, the author also reported a lesser LMA insertion time in the standard technique (22.5±3.1 seconds) than in the reverse technique (25.5±7.8 seconds). The mean difference was statistically significant (p-value: 0.0001). Similar results were also seen by Haghighi et al. [17].

Consistent with our study, Prasad et al. conducted a randomized, prospective, single-blinded study and compared the standard technique to the reverse technique of classic LMA insertion in 60 adult patients [16]. They reported that the mean insertion time taken was 12.08 (range of 8.53-17.21) seconds in the standard technique group and, in the reverse technique group, 13.99 (range of 8.37-24.08) seconds. The similarity in our study insertion time for a successful airway in the standard technique group was less. In concordance with our study, Keerthana et al. conducted a prospective randomized study for the evaluation of two insertion techniques of LMA insertion, standard and 18-degree rotation in adult patients [18]. And they reported a lesser LMA insertion time in the standard technique (24.79±4.19 seconds) than in the reverse technique (26.73±33.97 seconds). The mean difference was statistically insignificant (p-value: 0.10).

Our findings revealed no significant difference in the incidence of complications between the two groups. Similar results were seen in the study conducted by Keerthana et al. in terms of complications [18]. Their study concluded that the incidence of postoperative sore throat and bleeding was similar between the two groups, and no statistically significant difference was noted (p=0.40 and p=0.25, respectively). Consistent with our findings, Balaji and Jayaprakash observed no statistically significant variation in the incidence of complications when comparing the standard to rotational LMA insertion techniques [20].

Limitations

Operator variability and expertise in techniques might affect the procedural success rate, time of insertion, and complication rates. We used c-LMA in our study; different LMAs have different shapes, so it cannot be generalized for all the LMAs. Reusing the LMA up to 40 times can lead to difficulty in insertion with a different technique, though in our study, we have used the LMA up to a maximum of 20 times. Further studies can be conducted with a higher sample size and in different centers for the generalizability of our study results.

## Conclusions

We concluded that in the pediatric population, the standard technique of LMA insertion serves as a more effective and practical alternative compared to the reverse technique. The superiority of the standard technique may be attributed to its shorter insertion time, easier maneuverability, and consistent success rate while maintaining a comparable safety profile with minimal complications. These findings highlight the advantage of using the conventional approach, particularly in situations where rapid airway management is essential. Despite these findings, the role of the reverse technique cannot be disregarded in situations where difficulty is encountered while negotiating the tongue. However, the choice of insertion technique ultimately depends on the individual anesthesiologist's preference and experience.
